# Molecular Evolution and Structural Mapping of N-Terminal Domain in Spike Gene of Middle East Respiratory Syndrome Coronavirus (MERS-CoV)

**DOI:** 10.3390/v12050502

**Published:** 2020-05-02

**Authors:** Asif Naeem, Maaweya E. Hamed, Majed F. Alghoribi, Waleed Aljabr, Hadel Alsaran, Mushira A. Enani, Bandar Alosaimi

**Affiliations:** 1 Department of Research Laboratories, Research Center, King Fahad Medical City, Riyadh 59046, Saudi Arabia; anaeem@kfmc.med.sa (A.N.); waljabr@kfmc.med.sa (W.A.); halsaran@kfmc.med.sa (H.A.); 2 Department of Microbiology, College of Science, King Saud University, Riyadh 11451, Saudi Arabia; mawdalla@ksu.edu.sa; 3 Infectious Diseases Research Department, King Abdullah International Medical Research Center, Riyadh 11481, Saudi Arabia; alghoribima@ngha.med.sa; 4 Medical Specialties Department, King Fahad Medical City, Riyadh 59046, Saudi Arabia; menani@kfmc.med.sa; 5 Department of Basic Medical Sciences, College of Medicine, King Fahad Medical City, Riyadh 59046, Saudi Arabia

**Keywords:** Middle East Respiratory Syndrome Coronavirus, evolution, viral load, spike gene

## Abstract

The Middle East Respiratory Syndrome Coronavirus (MERS-CoV) is a lethal zoonotic pathogen circulating in the Arabian Peninsula since 2012. There is no vaccine for MERS and anti-viral treatment is generally not applicable. We investigated the evolution of the MERS-CoV spike gene sequences and changes in viral loads over time from patients in Saudi Arabia from 2015–2017. All the MERS-CoV strains belonged to lineage 5, and showed high sequence homology (99.9%) to 2017 strains. Recombination analysis showed a potential recombination event in study strains from patients in Saudi Arabia. The spike gene showed eight amino acid substitutions, especially between the A1 and B5 lineage, and contained positively selected codon 1020. We also determined that the viral loads were significantly (*p* < 0.001) higher in fatal cases, and virus shedding was prolonged in some fatal cases beyond 21 days. The viral concentration peaked during the first week of illness, and the lower respiratory specimens had higher levels of MERS-CoV RNA. The presence of the diversifying selection and the topologies with the structural mapping of residues under purifying selection suggested that codon 1020 might have a role in the evolution of spike gene during the divergence of different lineages. This study will improve our understanding of the evolution of MERS-CoV, and also highlights the need for enhanced surveillance in humans and dromedaries. The presence of amino acid changes at the N-terminal domain and structural mapping of residues under positive selection at heptad repeat 1 provides better insight into the adaptive evolution of the spike gene and might have a potential role in virus-host tropism and pathogenesis.

## 1. Introduction

Middle East Respiratory Syndrome (MERS) was discovered in a patient who died from acute respiratory illness in Jeddah, Saudi Arabia in 2012. A novel coronavirus (MERS-CoV) was isolated from this patient [1]. The virus was initially designated as HCoV (human coronavirus)-Erasmus University Medical Center (EMC), but after consensus, it was renamed MERS-CoV [2]. The retrospective analysis identified MERS-CoV from an outbreak involving 13 individuals in 2012 in Jordan [3]. MERS cases were reported from the Arabian Peninsula, Asia, Europe, Africa, and the USA. Since September 2012, the World Health Organization (WHO) has notified over 2494 laboratory-confirmed cases of MERS-CoV infection, with more than 850 deaths [4]. Saudi Arabia has reported the majority of the cases (as of 24 December 2019, 2102 total laboratory-confirmed cases, and 780 deaths (37.1% mortality) [5].

Coronaviruses are large (28–32 kb) single-stranded positive sense RNA viruses [6]. MERS-CoV belongs to lineage c beta-coronaviruses. The genomic structure of MERS-CoV has been explained with proteins involved in virus replication that are encoded at the 5′ end, and structural proteins expressed from the genes at the 3′ end of the genome [7]. The functional receptor for MERS-CoV is dipeptidyl peptidase 4 (DPP4 or CD26); it is present on the surface of human non-ciliated bronchial epithelial cells [8,9]. The receptor-binding domain (RBD) consists of residues E382 to C585 [10,11].

Due to the high number of cases in Saudi Arabia, there is a chance that MERS-CoV might have acquired mutations to become more virulent and transmissible. However, viruses isolated during the outbreaks showed no signs of any change in replication, immune evasion, interferon sensitivity, or neutralization compared to the circulating strains or the original virus from 2012 [12]. The clinical manifestations among hospitalized MERS-CoV patients include severe acute respiratory illness, sometimes associated with hypoxic respiratory failure and extra-pulmonary organ dysfunction [13]. Mild and asymptomatic infections were identified through contact tracing [14,15].

Recombination has been known to be common among beta-coronaviruses [16,17]. New viral strains emerge due to recombination events by joining the previously unlinked RNA. These strains could have a better host range and are able to evade the immune responses of the host. The phylogenetic analysis of the MERS-CoV has shown the presence of two clades (clade A and B), and clade B has five phylogenetic groups or lineages [18]. It has been documented that the recombination event took place between lineage 3 and lineage 5 [18]. However, there is little evidence of potential recombination events between the rest of lineages.

Multiple animal-to-human introductions of MERS-CoV have been analyzed in Saudi Arabia from 2012–2013 due to genetic heterogeneity [19]. There is evidence of the introduction of several strains, even in single hospital outbreaks [12,20,21]. The number of MERS-CoV cases increased in 2014, then declined later. However, sporadic cases continue to be detected [21]. A large outbreak occurred in South Korea in mid-2015, which was originally due to an importation from the Arabian Peninsula [22]. Case clustering of MERS in the UK, Italy, Tunisia, France, Iran, South Korea, the United Arab Emirates, and healthcare facilities in Saudi Arabia strongly suggests human-to-human transmission [12,13,23,24,25,26,27,28]. The number of contacts infected with confirmed infections, however, appears to be limited [29]. It raises the possibility that the number of previously infected individuals in Saudi Arabia is much greater than documented and necessitates the efforts to increase the genetic characterization of MERS-CoV suspected cases.

We report the investigation of MERS cases and molecular evolution of MERS-CoV Spike (S) genes isolated from patients during 2015–2017 on the basis of variation and phylogenetic analysis along with viral load kinetics.

## 2. Materials and Methods

### 2.1. Ethical Considerations

Ethics approval and consent to participate: All procedures performed in this study involving clinical specimen were in accordance with the ethical standards of the institutional and national research committee and with the 1964 Helsinki Declaration and its later amendments or comparable ethical standards. The study was approved by the Institutional Review Board at King Fahad Medical City (IRB Log No. 16-346 approved on 27 September 2016). Informed consent to participate was waived or not required since only remaining left-over specimens were used for this study.

Availability of data and materials: The clinical datasets used and analyzed during the current study are available from the corresponding author on request.

### 2.2. Biosafety Considerations

The handling of respiratory samples, as well as preparation of aliquots and viral RNA extraction, was performed using appropriate personal protective equipment in the biosafety level 3 laboratory (Riyadh Regional Laboratory, Ministry of Health, Riyadh, Saudi Arabia).

### 2.3. Study Specimens

The study specimens were obtained from a population (*n* = 13) admitted to various hospitals in Riyadh, Saudi Arabia with six associated deaths. All samples (*n* = 40) testing positive for MERS-CoV locally by real-time reverse transcription PCR (rRT-PCR) assay and admitted to the hospital from 1 September 2015–16 November 2017, were used for this study (Table 1). Written informed consent was obtained from the patients at the time of admission.

### 2.4. Laboratory Investigations

Nasopharyngeal swab specimens, tracheal aspirates, sputum, or bronchoalveolar lavages were collected for laboratory-based investigations. A case of MERS, according to the Saudi Arabian Ministry of Health definition, is fever and acute respiratory illness in a patient who has a positive test result for MERS-CoV infection. Demographic information, medical history, and outcome information were collected. Respiratory specimens were used for upE [30] and ORF1a rRT-PCR assays [31] for MERS-CoV initial screening and further molecular testing.

### 2.5. RNA Extraction

RNA was extracted from the samples as described earlier [32] using a viral RNA mini kit (Qiagen). A 2× sputum lysis buffer (10 g of *N*-acetylcysteine/liter, 0.9% *w*/*v* sodium chloride) was used for the treatment of sputum samples for 30 min in a shaking incubator. Swabs were dissolved in the lysis buffer.

### 2.6. Real-Time PCR Assay—Upstream of E Gene (upE Assay)

A 25-μL reaction was prepared containing 5 μL of RNA, as described previously [30]. Briefly, 12.5 μL of 2× reaction buffer provided with the Superscript III one-step RT-PCR system with Platinum Taq Polymerase (Invitrogen, Carlsbad, CA, USA), 1 μL of reverse transcriptase/Taq mixture from the kit, 0.4 µL of a 50 mM magnesium sulfate solution (Invitrogen, Carlsbad, CA, USA), 1 μL each of 10 μM forward and reverse upE primers with 400 nM concentration in the final solution, as well as 0.5 μL of 10 μM upE probe with 200 nM concentration in final solution was used. Molecular grade deionized water was used to make the final volume to 25 μL. Thermal cycling involved reverse transcription at 55 °C for 20 min, followed by denaturation at 95 °C for 3 min, and then 45 cycles of denaturation and extension at 95 °C for 15 s and 58 °C for 30 s.

### 2.7. Confirmatory Real-Time PCR Assay in ORF 1A (1A Assay)

A 25 µL reaction was prepared containing 5 µL of RNA, as described previously [31]. Briefly, 12.5 µL of 2× reaction buffer from the Superscript III one-step RT-PCR system with Platinum Taq Polymerase (Invitrogen, Carlsbad, CA, USA), 1 µL of reverse transcriptase/Taq mixture from the kit, 0.4 µL of a 50 mM MgCl_2_ solution (Invitrogen, Carlsbad, CA, USA), 1 μL each of 10 μM forward and reverse of EMC-Orf*1a* primers with 400 nM concentration in the final solution, as well as 0.5 μL of 10 μM EMC-Orf*1a* probe with 200 nM concentration in the final solution was used. Molecular grade deionized water was used to make the final volume to 25 μL. Thermal cycling involved reverse transcription at 55 °C for 20 min, followed by denaturation at 95 °C for 3 min, and then 45 cycles of denaturation and extension at 95 °C for 15 s, and 58 °C for 30 s.

### 2.8. Genetic Sequencing & Phylogenetic Analyses

Extracted viral RNA was reverse transcribed using random hexamers (Invitrogen, Carlsbad, CA, USA) at 52 °C for 60 min with Superscript III reverse transcriptase (Invitrogen, Carlsbad, CA, USA) by following the manufacturer’s instructions. Five μL of the cDNA was amplified by PCR using MERS-CoV specific primer sets with the Phusion Flash High Fidelity PCR master mix kit (Thermo Scientific, Carlsbad, CA, USA). PCR cycling conditions were as follows: an initial activation step at 95 °C for 15 min, followed by 35 cycles of 95 °C for 1 min, 55 °C for 1 min, and 68 °C for 2 min, with a final extension of 68 °C for 10 min on a Veriti PCR system (Applied Biosystems, Thermo Fisher Scientific, Waltham, MA, USA). Amplified products were purified with the ExoSAP-IT Express PCR Cleanup Reagent (Thermo Fisher Scientific, Waltham, MA, USA), the nucleotide sequences of the amplified fragments from study isolates, with high copy numbers, were determined using M13-tailed PCR primers and BigDye Terminator v3.1 Cycle Sequence kit according to the manufacturer’s instructions (Applied Biosystems, San Diego, CA, USA). Removal of unincorporated ddNTP’s, salts, and dye blobs were performed by BigDye XTerminator Purification Kit (Applied Biosystems, San Diego, CA, USA). Capillary electrophoresis was performed in SeqStudio Sanger sequencer (Applied Biosystems, San Diego, CA, USA), and analysis of Sanger sequencing data, by assembly and editing, was conducted by Sequencher 5.4.6 (Gene Codes Corporation, Ann Arbor, MI, USA). Nucleotide sequences, except primer regions, were aligned with those retrieved from the Genbank Nucleotide Sequence Database. Inspection and manual modification and evolutionary analyses were conducted using Molecular Evolutionary Genetics Analysis Version X (MEGA-X), phylogenies were estimated using neighbor-joining and maximum likelihood methods implemented in MEGA, version X [33]. The neighbor-joining method used maximum composite likelihood distance estimation, and maximum likelihood used the Tamura–Nei (TN93) model of nucleotide substitution with γ-distributed rate variation (TN93 + G) and bootstrapping (1000 replicates). The genomic data was analyzed and submitted to a public database (GenBank) under accession numbers MK858156-MK858164 and MN735679-MN735682 (Supplementary Table S1).

### 2.9. Recombination Analysis and Phylogenetic Reconstruction Based on Recombinant Fragments

Potential recombination events among MERS-CoV S gene complete reference sequences retrieved from GenBank sequence database (*n* = 147) and the study strains (*n* = 13) (Supplementary Table S1) were identified using automated RDP, GENECONV, BOOTSCAN, MaxChi, CHIMAERA, and SISCAN methods provided in RDP4 software [34]. Moreover, the evidence of recombinant breakpoint was confirmed using GARD in Datamonkey web server [35]. Since recombination can affect phylogenetic tree inferences [36], we followed the procedure presented in [37]. Thus, a phylogenetic tree based on each recombinant fragment was reconstructed after selecting the TN93 + G model with the lowest BIC score using the MEGA software version X [33].

### 2.10. Measurement of Selection Pressure

The selective pressure on encoding S gene of MERS-CoV was examined by calculating the ratio of synonymous and non-synonymous substitutions (d*N*/d*S*, defined as ω) across lineage on a codon-by-codon basis. The individual site-specific selection pressure and ω were estimated using the single likelihood ancestor counting (SLAC), fixed effects likelihood (FEL), and mixed-effects model of evolution (MEME) methods contained in the HYPHY package [35]. All analyses utilized the Datamonkey online tool (http://www.datamonkey.org). The value of ω was estimated based on the neighbor-joining trees under the GTR substitution model. The significance level for a positively selected site by either SLAC/FEL/MEME or all three methods was accepted at 0.1.

### 2.11. Prediction of Glycosylation Sites

The NetNGlyc 1.0 server was used to predict the potential N-linked glycosylation sites (amino acids Asn-X-Ser/Thr, whereby X is any amino acid except for Asp or Pro) [38]. A threshold value of >0.5 for the average potential score suggests glycosylation.

### 2.12. MERS-CoV Viral Load Assay

To measure the viral load, a one-step multiplex quantitative rRT-PCR was performed with RealStar^®^ MERS-CoV (upE & ORF1a) RT-PCR Kit (Altona Diagnostics, Hamburg, Germany). All assays were performed in triplicate with the 7500 Fast Real-time PCR system (Applied Biosystems^®^, Grand Island, NY, USA). Positive controls of known concentrations of 1.0 × 10^5^ copies per μL were kindly provided by the European Virus Archives (EVA). Each sample was tested in triplicate, and the mean of the three values was shown as the copy number of the sample. Samples were defined as negative if the copy number fell below 10. The copy number was calculated based on the standard curve generated by real-time RT-PCR using the control RNA and expressed as copies/mL.

### 2.13. Statistical Analysis

The independent *t*-test was used to assess the statistical significance of differences between groups. In all analyses, *p* < 0.05 was used to indicate statistical significance. Viral RNA copy number was compared between groups (dead and recovered) by Mann–Whitney U test, and the proportion of cases with detectable RNA was compared using Fisher’s exact test. Demographic and clinical characteristics were analyzed, and differences assessed for significance by using Chi-square test, Wilcoxon rank-sum, and Kruskal–Wallis tests where appropriate. Statistical analyses were performed using the IBM SPSS Statistics software (version 22.0, IBM Corporation, Armonk, NY, USA).

## 3. Results

**Figure 1:**
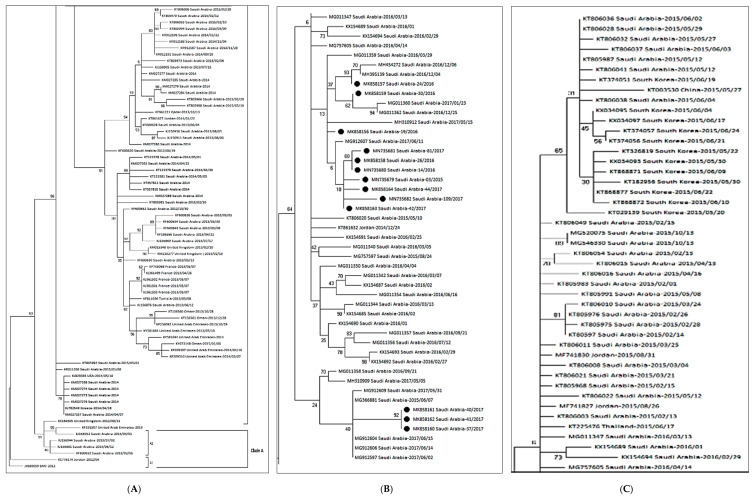
The maximum likelihood phylogenetic tree based on S gene sequences of 160 MERS-CoV strains is shown. The TN93 + G substitution model was used to generate the S gene tree. Representative strains for clade A are shown in the Figure 1A. Clade b is split into three sections (**A**–**C**) for better Lineage 5 is enlarged with annotations to clarify the relationship of the present study strains (**B**). Bootstrap values in percentage are shown next to the branches. The scale bar indicates the number of nucleotide substitutions per site. Selected MERS-CoV strains were annotated. The 13 MERS-CoV strains from the present study are indicated in black circles (**B**).

The 160 complete S gene sequences of MERS-CoV clustered into two major clades, A and B (Figure 1). Clade A had only five strains, while most of the strains clustered into clade B. Clade A has historically been divided into two lineages; lineage A1, containing the first reported MERS-CoV strain from Saudi Arabia in 2012 and two Jordan strains. While lineage A2 contained two camel strains from the United Arab Emirates (UAE) and Egypt. Clade B had a further six lineages containing human strains. Lineage B2 to B6 mostly contained the Saudi strains. B3 lineage was mostly populated by the South Korean strains, and the same strain carried to various countries like Bahrain, Qatar, China, Saudi Arabia, and the UAE during 2015.

A comparative genetic analysis of 160 strains isolated in 27 countries, including Saudi Arabia, demonstrated that the circulating 13 MERS-CoV strains from our study showed the highest identity (99.9%) with the strains recently isolated in Saudi Arabia [39,40,41]. Based on the respiratory specimen available, S gene sequences from 13 patients appeared to have a close relationship with each other and belonged to lineage 5 [42].

There were three amino acid substitutions in the N terminal domain (NTD) of the S gene in the same comparison (Table 2). The 2017 strains from our study, i.e., MK858160-Saudi Arabia-37-2017, MK858161-Saudi Arabia-40-2017, and MK858162-Saudi Arabia-41-2017 had three amino acid substitutions at S126A, F228L, and L745F, respectively. The only 2015 strain (MN735679-Saudi Arabia-03-2015) from our study also had a single mutation at S126A. In the phylogenetic analysis, the virus was located in a lineage of locally endemic 2016 and 2017 Saudi Arabia strains (lineage 5 in Figure 1) but was distinguished from 2015 strains.

**Figure 2:**
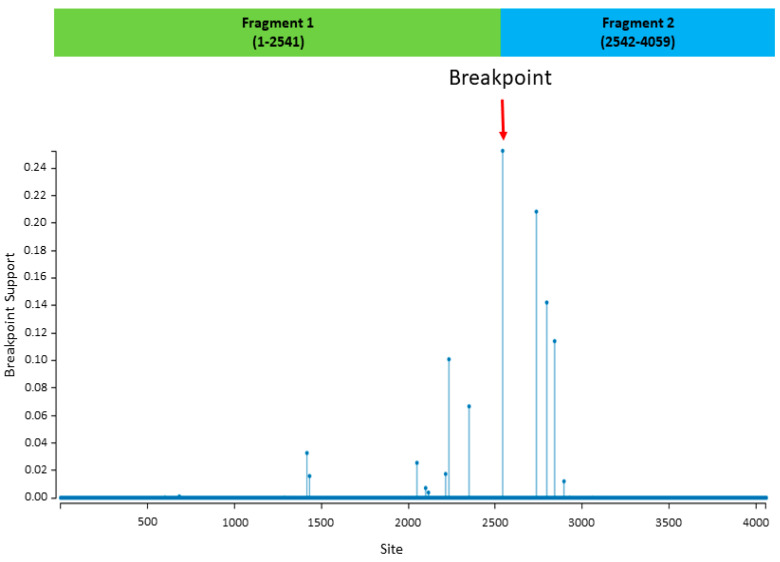
Results of recombination analysis using genetic algorithm for recombination detection (GARD) in the Datamonkey web server. The red arrow indicates a significant breakpoint that was detected (*p*-value < 0.05). The *x*-axis represents the nucleotide site. The two recombinant fragments are presented in the green and blue bars.

The recombination analysis showed that there was one significant (*p*-value ≤ 0.05) event related to the study strain 19 (major parent) and the reference strain from clade A (minor parent) that was detected by all recombinant analytical methods (RGBMCST) with all automated runs available in RDP4. The recombination detection program (RDP) plot and genetic algorithm for recombination detection (GARD) showed that the recombination occurred between nt positions 1–2541 of the major parent, and 2542–4059 of the minor parent (Figure 2). This breakpoint generated two recombinant fragments indicating statistically significant incongruence in lineage 5. The first fragment (aa 1–848) involves the S1 subunit with NTD, RBD, Sub-domain 1 and 2, followed by a linker region. The second fragment (aa 848–1353) involves almost the entire S2 subunit regions. Additional phylogenetic analyses were separately performed based on two recombinant fragments. The results of the phylogenetic analyses demonstrated that, based on the recombinant fragments, the Saudi strains were grouped together with the parent strain, separated from MERS-CoV from other countries (data not shown). The results of the phylogenetic analyses, based on each recombinant fragment, confirmed the findings of recombination events generated from GARD (Figure 2).

### 3.1. Selective Pressure Analysis

We assessed the adaptive evolution of MERS-CoV, d*N*/d*S* ratios (ω) in the S gene across the 13 strains with codon-by-codon basis calculations. The S gene was expected to be under selection pressure to escape the host immune responses; therefore, the rate of change was determined by *ω* values where *ω* < 1 indicated the presence of negative or purifying selection, neutral selection pressure was indicated by *ω* = 1, and positive or diversifying selection was shown by *ω* > 1 [43]. The analysis showed that the overall ω was 0.1741737, with most of the codons having ω < 1, indicating purifying selection. However, the codon 1020 had an *ω* value of more than 1 by the mixed-effects model of evolution (MEME) method with statistical significance, which indicated the presence of functional constraints during the evolutionary process. Strains belonging to clade B were predicted to have codon 1020 and 1267 under positive selection. Amino acid residues in the receptor-binding domain (RBD) remained conserved and were not found to be under selection pressure.

### 3.2. Prediction of Glycosylation Sites

The majority of MERS-CoV strains possessed three potential glycosylation sites in the S1 subunit at amino acid positions 135 and 206 in the NTD region, and S2 subunit at amino acid position 1243 which were present in all lineage B5 strains. We did not find any mutations resulting in a loss of potential glycosylation site especially in the RBD region.

### 3.3. Modeling of Spike Protein

**Figure 3:**
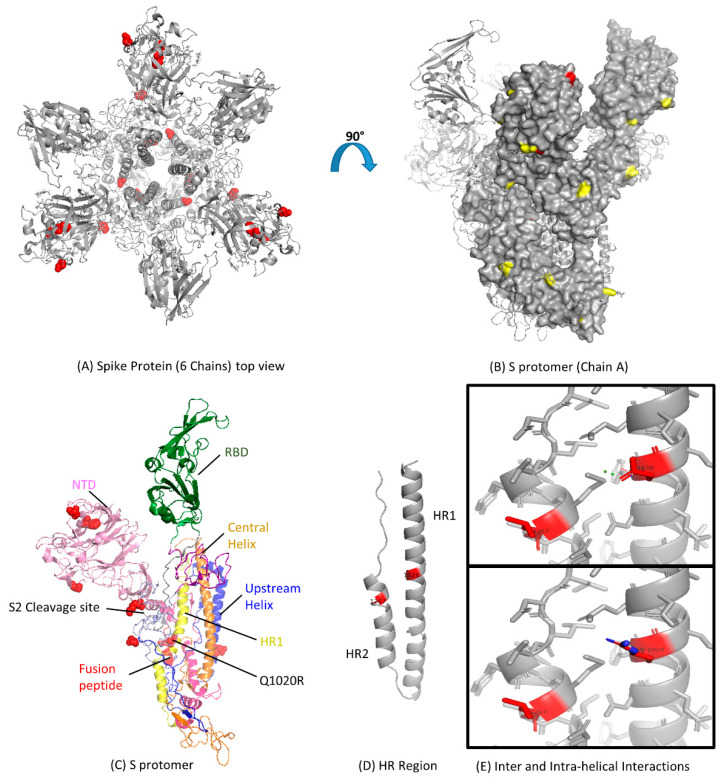
Functional domains and structural view of amino acid substitutions in the S gene of Middle East Respiratory Syndrome Coronavirus (MERS-CoV). (**A**) Ribbon representation of the MERS-CoV S gene with amino acid substitutions shown in red spheres with resolved structure in protein database identification (PDB ID: 5 × 59); (**B**) Surface representation of the S protomer in the Chain A side view showing known glycosylation sites in yellow with amino acid substitutions in red color (PDB ID: 5 × 59); (**C**) Cartoon representation of all the S protomer for detailed architecture with amino acid changes shown in red spheres; NTD, N-terminal domain; RBD, receptor-binding domain; HR, heptad repeat; CH, central helix (PDB ID: 5 × 59); (**D**) Ribbon representation of HR region of S protomer; Amino acid changes Q1020R and L1267S are shown in red color (PDB ID: 4MOD); (**E**) Structural mapping of residue 1020 in the form of inter and intra-helical interactions. The top box shows the interactions for the glutamine residue (Q1020), and the lower box shows the arginine residue (R1020). Hydrogens were removed for a better view. The figures were produced using PyMOL.

The models of S gene from each clade were built. We found that the amino acid substitutions observed by multiple alignments appeared to be randomly distributed throughout the structure (Figure 3A). The MERS-CoV S gene has 25 potential N-linked glycosylation sites (Figure 3B). Most of the N-linked glycosylation sites are located on the S1 subunit and the C-terminal region (including the HR2 region and the region preceding HR2) of the S2 subunit (Figure 3B). For FR, the HR1 region, and the central helix, there are no N-linked glycosylation sites (Figure 3C,D). Based on the homology 3D crystal structure model, no specific evolutionary pattern was found in the MERS-CoV S gene. However, we found that the codon 1020 residue could differentiate between clade A and B (Figure 3C,E). Clade A strains have a glutamine at codon 1020 (Q1020), while clade B strains have an arginine residue (R1020). These results taken together, with the selection pressure analysis, suggest that codon 1020 might have a role during the evolutionary process of MERS-CoV S gene and the diversity of lineages in clade B.

### 3.4. Analysis of MERS-CoV HR Variation

After translation, the 180-kDa oligomeric S gene of the coronaviruses is cleaved into an S1 receptor-binding unit and an S2 membrane fusion unit [44]. S2 subunit contains an internal fusion peptide and two heptad regions (HR1 and HR2) (Figure 3D). HR2 is present at the membrane anchor, while HR1 is 170 amino acids upstream. The structure of the HR region of the MERS-CoV has been solved through its side chain [39]; we observed glutamine residue replacing arginine (observed in human-derived viruses) at 1020 position (Figure 3E). It might have led to the loss of side-chain interactions with M1266 due to another substitution at position 1267 in the HR2 region. That might also affect the hydrogen bond with the HR1 heptad and its stability.

### 3.5. Viral Loads Assay

**Figure 4:**
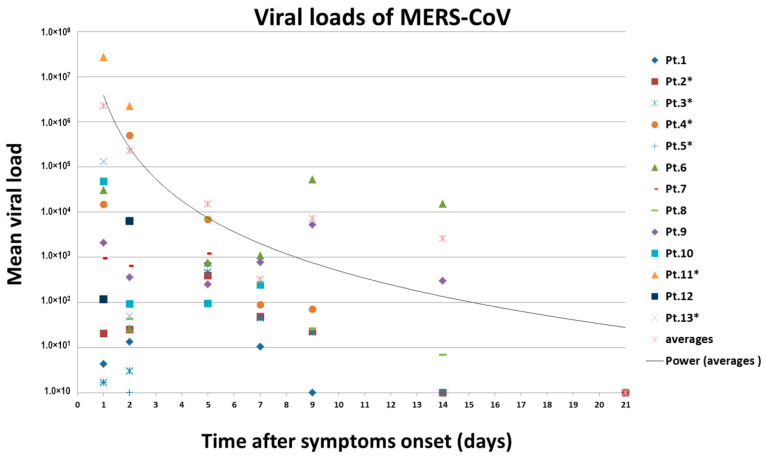
Changes in MERS-CoV viral RNA concentrations in respiratory samples over time in 13 patients. Estimated viral loads in respiratory tract specimens collected from hospitalized MERS-CoV patients, Saudi Arabia, 1 September 2015–16 November 2017. Patient numbers with an asterisk indicate the fatality. The RNA copies of MERS-CoV in a throat swab or tracheal aspirate were estimated by means of a standard curve in the upstream of envelop (upE) real-time reverse-transcriptase polymerase chain reaction. Different color–symbol combinations denote individual patients; data points below the limit of detection are shown at different levels for clarity.

A total of 13 patients’ throat swab and nasal swab specimens were analyzed in this study. The median incubation period was 12 days (range 0 to 32). Six patients died, out of which three patients were admitted to the hospital after almost three weeks of disease onset in 2015. The remaining fatal cases were admitted to the hospital during the first week of disease onset with highly significant (*p* < 0.001) viral loads (nasal swabs and tracheal aspirate specimens) in 2017 (Figure 4). The highest viral loads, in terms of MERS-CoV RNA copies detected by rRT-PCR in respiratory specimens, were observed during the first week of disease onset. The median value was 6.91 log_10_ copies per milliliter among the patients that died, and 5.25 log_10_ copies per milliliter among the patients that recovered (*p* = 0.05). The peak in viral load of tracheal aspirates was higher and occurred earlier in patients that died than recovered patients (Figure 4). RNA concentration exceeded 10^7^ copies per milliliter in samples from patients that died except the other three patients in the same group. On the other hand, RNA concentration did not exceed 10^4^ copies per milliliter in samples from patients that recovered (*p* < 0.001).

## 4. Discussion

We determined the S gene sequences from 13 patients and conducted the phylogenetic analysis to determine the genetic relationship and heterogeneity between 13 Saudi and 147 reference MERS-CoV strains from humans. The evolution of clade A and clade B MERS-CoV based on S gene sequences was considerably different from each other. Historically clade B MERS-CoV has out-numbered clade A MERS-CoV strains (Figure 1). This might be due to the transmission advantage of clade B MERS-CoV or sampling bias. The presence of strains from a single country like Saudi Arabia or UAE in various lineages indicates the sporadic transmission from dromedaries to humans with fatal outcome in some cases.

Lineage B5 divergence and separate evolution from other clade B lineages might have caused the number of mutations in the S gene compared to lineage A2. This was shown by the deep branching pattern of lineage B5 in the phylogenetic tree, as well as a change of amino acids at different positions of the S1 and S2 subunit of the S gene (Table 2). The residue at 1020 is known to distinguish between clade A and B strains [39], and it was positively selected among all the strains from our study, suggesting a role in the divergence of different lineages during the evolution of the S gene. This positive selection of codon 1020 suggests that the evolution of the Saudi strains was a genetic selection, and the virus was not imported, rather it continuously evolved in the population. However, further studies are required to determine the role of this mutation in the survival of lineage B strains over lineage A.

The recombination analysis suggested one potential recombination event involving lineage 5. Absence of additional recombination events meant that double infection and super-infection might not have been present during the transmission of MERS-CoV. We could not detect any potential large recombination fragments among lineage 2, 3, and 5, as previously known [45]. That could be resolved by continued identification and characterization of circulating MERS-CoV strains and developing a better-detailed classification system for various MERS-CoVs in the future. 

We detected the positive selection in the S gene of MERS-CoVs, which indicates that they probably experienced strong adaptive evolutionary pressure from the host’s immune system. The phylogenetic reconstruction analyses have established previously [42] that lineage 5 MERS-CoVs evolved from a recombinant virus composed of lineage 3 and lineage 4 viruses. The recombinant lineage 5 viruses were shown to have acquired the 5′ part of ORF1ab and the 3′ part of the S gene from lineage 4 and the remaining genomic regions from lineage 3. In our S gene-based phylogenies, lineage 5 viruses were closely related to lineage 3 and 4 viruses from Saudi Arabia. One of the limitations of our study was the availability of complete S gene sequences only, instead of the complete genome sequences, which did not allow us to look for amino acid substitutions in other regions, especially the non-structural proteins of the ORF1ab polyprotein.

The glycan-binding site on the top of MERS-CoV NTD is covered by a short helix along with the N-linked glycan. Unlike BCoV and HKU1, this conformational change in NTD might make it unable to attach the cell surface by recognizing certain sugar molecules [46,47]. The S1 subunit of MERS-CoV also contains two subdomains (I and II) along with NTD and RBD domains. These subdomains appear to support the NTD and RBD domains (Figure 3B). The S2 subunit forms the stem region of the S gene and is mainly composed of α-helices (Figure 3B). The S2 cleavage site is connected to the long upstream helix by a long linker region that exposes it to the peripheral side, and thus, becomes a target for endo-lysosomal proteases (Figure 3B). The helical fusion peptide is also exposed beneath the S2 cleavage site and provides a connection between the HR1 region involving three consecutive α-helices. A long central helix follows along the three-fold axis towards the viral membrane from the HR1 region (Figure 3C). The 3-D structure of the MERS-CoV S gene is very similar to influenza virus hemagglutinin (HA) protein, which also has two subunits (HA1 and HA2) [48]. Similarly, the HA1 subunit separates from HA2 before membrane fusion starts under a low pH environment in the endosome [49].

We found amino acid substitutions in the NTD region, which is one of the major functional sites of the S gene [50]. In addition, the strains involved in the present study had nine amino acid substitutions in the S1 and S2 subunits of S gene. The outbreak in South Korea in 2015 had 12 nt substitutions (NTD, 1 site; RBD, 2 or 3 sites), implying that the strains involved diverged from a different virus [51]. MERS-CoV strains previously associated with outbreaks in Korea and other neighboring countries were most closely related (99.9% nucleotide identity) to a human MERS-CoV strain isolated in Riyadh, Saudi Arabia, in 2017. Furthermore, these strains had five nucleotide substitutions (C309T, T519C, C2928T, T3375C, and T3598C) compared with a MERS-CoV strain found in Riyadh in 2017 [50].

Coronaviruses are known to undergo recombination [52], and it is considered to be related to an increase in pathogenicity in other animal RNA viruses [53]. There is, however, little evidence that recombination in MERS-CoV has caused substantial changes in human-to-human transmission. At the same time, recombinant MERS-CoVs have been circulating in Saudi Arabia in camels [42] and humans since 2015 [21,54]. However, these circulating variants have not caused any major change in human CoV epidemiology [21]. There has also been a report about the circulation of deletion variants of MERS-CoV among humans in Jordan [55], again without any major changes in epidemiology [56].

We observed that the viral loads were higher in the fatal cases (Pt.11, Pt.12, and Pt.13) and the patients that survived (Pt.6 and Pt.9) had more prolonged viral shedding in respiratory secretions (throat swabs). Previous studies involving blood viral RNA had low detection rates in the early phase of infection in patients with confirmed diagnosis [57,58]. These findings, however, are similar to studies involving the MERS-CoV outbreak in Saudi Arabia [59], Korea [60], and severe acute respiratory syndrome (SARS) [61].

In summary, we obtained genetic information about the circulating MERS-CoV strains in Saudi Arabia from 2015 to 2017, belonging to lineage 5. Codon 1020 was positively selected suggesting that this residue might have played a role in the evolution of the S gene during the divergence of different lineages. The MERS-CoV RNA concentration were highest during the first week of illness and lower respiratory tract specimens had higher levels than upper respiratory tract specimens. We hope that our genetic and viral loads data would support further epidemiological investigations of MERS-CoV. It will also add to our current understanding of the evolutionary process of the S gene of MERS-CoV and a need for better surveillance in humans and dromedary camels. The presence of amino acid changes at the N-terminal domain and the structural mapping of residues under positive selection at heptad repeat 1 provides better insight and might have a potential role in virus-host tropism and pathogenesis.

## Figures and Tables

**Table 1:** Clinical and epidemiological profile of Middle East Respiratory Syndrome Coronavirus (MERS-CoV) cases.

Patient No.	Sample No.	Gender	Age	Specimen Type *	Onset Date	Date of Sample Collection	E-Gene Ct	Orf1a Ct	Health-Care Worker	Outcome
1	1	Female	57	NPA	20-Sep-15	23-Sep-15	37	35	NO	Recovered
	2	Female	57	NPA	20-Sep-15	24-Sep-15	34	35		
	3 **	Female	57	NPA	20-Sep-15	28-Sep-15	30	30		
	4	Female	57	NPA	20-Sep-15	29-Sep-15	35	36		
2	1	Female	59	NPA	30-Aug-15	23-Sep-15	35	36	NO	Died
	2 **	Female	59	NPA	30-Aug-15	28-Sep-15	32	32		
	3	Female	59	NPA	30-Aug-15	29-Sep-15	34	33		
	4	Female	59	NPA	30-Aug-15	01-Oct-15	35	36		
3	1 **	Male	57	NPA	10-Sep-15	29-Sep-15	33	33	NO	Died
	2	Male	57	NPA	10-Sep-15	01-Oct-15	37	37		
4	1	Female	75	NPA	12-Sep-15	30-Sep-15	30	30	NO	Died
	2 **	Female	75	NPA	12-Sep-15	02-Oct-15	28	29		
5	1	Male	26	NPA	30-Jun-16	02-Jul-16	25	26	NO	Died
	2 **	Male	26	NPA	30-Jun-16	04-Jul-16	22	22		
6	1 **	Male	26	NPA	04-Jun-17	07-Jun-17	26	26	YES	Recovered
	2	Male	26	NPA	04-Jun-17	16-Jun-17	34	36		
	3	Male	26	NPA	04-Jun-17	19-Jun-17	30	30		
	4	Male	26	NPA	04-Jun-17	23-Jun-17	27	27		
	5	Male	26	NPA	04-Jun-17	25-Jun-17	26	27		
7	1 **	Female	27	NPA	08-Jun-17	08-Jun-17	27	27	YES	Recovered
	2	Female	27	NPA	08-Jun-17	13-Jun-17	30	30		
	3	Female	27	NPA	08-Jun-17	16-Jun-17	31	31		
8	1	Male	39	NPA	11-Jun-17	11-Jun-17	30	31	YES	Recovered
	2	Male	39	NPA	11-Jun-17	16-Jun-17	34	34		
	3 **	Male	39	NPA	11-Jun-17	19-Jun-17	27	27		
	4	Male	39	NPA	11-Jun-17	25-Jun-17	30	32		
	5	Male	39	NPA	11-Jun-17	29-Jun-17	32	32		
	6	Male	39	NPA	11-Jun-17	03-Jul-17	34	35		
9	1 **	Female	35	NPA	31-May-17	13-Jun-17	26	26	YES	Recovered
	2	Female	35	NPA	31-May-17	16-Jun-17	32	32		
	3	Female	35	NPA	31-May-17	19-Jun-17	29	30		
	4	Female	35	NPA	31-May-17	23-Jun-17	33	33		
10	1 **	Male	39	NPA	16-Jun-17	19-Jun-17	25	25	NO	Recovered
	2	Male	39	NPA	16-Jun-17	27-Jun-17	33	34		
11	1 **	Male	75	TA	22-Jun-17	23-Jun-17	15	15	NO	Died
	2	Male	75	TA	22-Jun-17	24-Jun-17	15	15		
12	1	Male	77	NPA	05-Oct-17	08-Oct-17	34	35	NO	Recovered
	2 **	Male	77	NPA	05-Oct-17	11-Oct-17	26	26		
13	1 **	Female	75	NPA	07-Nov-17	12-Nov-17	25	25	NO	Died
	2	Female	75	NPA	07-Nov-17	16-Nov-17	34	35		

* Nasopharyngeal Aspirate (NPA) and/or Tracheal Aspirate (TA); ** Samples with lowest Ct-value or highest RNA copy number were used for sequencing.

**Table 2:** Comparison of the S gene sequence variants of the MERS-CoV isolates from the patients in Saudi Arabia, 2015–2017, and reference strains.

	S1 Subunit											S2 Subunit				
	NTD						RBD						HR			
MERS-CoV Strains	32	95	126	200	222	228	473	556	588	700	710	745	848	1020	1224	1267
JX869059-England-2012/06/13 *	E	T	S	S	N	F	F	A	L	R	P	L	N	Q	G	L
MN735680-Saudi Arabia-14-2016														R		
MN735679-Saudi Arabia-03-2015			A											R		
MN735681-Saudi Arabia-81-2017														R		
MN735682-Saudi Arabia-109-2017													D	R		
MK858156-Saudi Arabia-19-2016														R		
MK858157-Saudi Arabia-24-2016										L				R		
MK858158-Saudi Arabia-26-2016														R		
MK858159-Saudi Arabia-30-2016				Y										R		
MK858160-Saudi Arabia-37-2017			A			L						F		R		
MK858161-Saudi Arabia-40-2017			A			L						F		R		
MK858162-Saudi Arabia-41-2017			A			L						F		R		
MK858163-Saudi Arabia-42-2017														R		
MK858164-Saudi Arabia-44-2017														R		S
KT806006-Saudi Arabia-2015 *					Y			V						R	S	
KT805971-Saudi Arabia-2015 *	A													R		
MG011342-Saudi Arabia-2016 *		I							I		H			R		
MG912606-Saudi Arabia-2017 *			A			L	S					F		R		

* Amino acid changes in the study sequences compared to the prototype sequences are shaded in gray. Amino acid abbreviations: Glutamic acid (E); Alanine (A); Threonine (T); Isoleucine (I); Serine (S); Tyrosine (Y); Asparagine (N); Phenylalanine (F); Leucine (L); Arginine (R); Proline (P); Aspartic acid (D); Glutamine (Q); Glycine (G).

## Supplementary Materials

Supplementary materials can be found at https://www.mdpi.com/1999-4915/12/5/502/s1. Table S1 Geographical and temporal distribution of 160 MERS-CoV isolates used for phylogenetic analysis.

## Abbreviations

MERS-CoV	Middle East Respiratory Syndrome Coronavirus
DPP4	dipeptidyl peptidase 4
RBD	Receptor binding domain
NTD	N terminal domain
HR1	Heptad repeat 1
S	Spike
Q	Glutamine
R	Arginine
RT-PCR	Reverse Transcription Polymerase Chain Reaction
KFMC	King Fahad Medical City
IRB	Institutional Review Board
