# Supplementary material for: Molecular Evolution and Structural Mapping of N-Terminal Domain in Spike Gene of Middle East Respiratory Syndrome Coronavirus (MERS-CoV)
Source: Viruses. 2020 May 2;12(5):502. doi: 10.3390/v12050502 (PMC7290774; doi:10.3390/v12050502)
Supplement: Supplementary file 1 [file viruses-12-00502-s001.pdf]

Table: Geographical and temporal distribution of 160 MERS-CoV isolates used for phylogenetic analysis.

Serial	Genbank Accession No.	Country	Year of Isolation
1	JX869059	The Netherlands	2012
2	MN735679	Saudi Arabia	2015
3	MN735680	Saudi Arabia	2016
4	MN735681	Saudi Arabia	2017
5	MN735682	Saudi Arabia	2017
6	MK858156	Saudi Arabia	2016
7	MK858157	Saudi Arabia	2016
8	MK858158	Saudi Arabia	2016
9	MK858159	Saudi Arabia	2016
10	MK858160	Saudi Arabia	2017
11	MK858161	Saudi Arabia	2017
12	MK858162	Saudi Arabia	2017
13	MK858163	Saudi Arabia	2017
14	MK858164	Saudi Arabia	2017
15	KT806006	Saudi Arabia	2015
16	KM027288	Saudi Arabia	2014
17	KT80597	Saudi Arabia	2015
18	KT861627	Jordan	2014
19	MG011342	Saudi Arabia	2016
20	KT868877	South Korea	2015
21	KT357811	Saudi Arabia	2014
22	KR912187	Saudi Arabia-	2014
23	KT156560	Oman	2013
24	KJ156881	Saudi Arabia	2013
25	KT805992	Saudi Arabia	2015
26	KX154689	Saudi Arabia	2016
27	KF745068	France	2013
28	KT029139	South Korea	2015
29	MG011340	Saudi Arabia	2016
30	KM027279	Saudi Arabia	2014
31	MG011350	Saudi Arabia	2016
32	KM015348	United Kingdom	2013
33	KX154687	Saudi Arabia	2016
34	KT806010	Saudi Arabia	2015
35	MF741827	Jordan	2015
36	KJ782549	Greece	2014
37	KF192507	United Arab Emirates	2013
38	MG912604	Saudi Arabia	2017
39	KT806021	Saudi Arabia	2015
40	KF600643	Saudi Arabia	2013
41	MG011357	Saudi Arabia	2016
42	KX154691	Saudi Arabia	2016
43	MG912606	Saudi Arabia	2017
44	KT121578	Saudi Arabia	2014

Serial	Genbank Accession No.	Country	Year of Isolation
45	KT805987	Saudi Arabia	2015
46	MG011358	Saudi Arabia	2016
47	KT121581	Saudi Arabia	2014
48	MG011359	Saudi Arabia	2016
49	MG011347	Saudi Arabia	2016
50	KP209307	United Arab Emirates	2014
51	KT805973	Saudi Arabia	2015
52	KT003530	Saudi Arabia	2015
53	MG011344	Saudi Arabia	2016
54	KT326819	South Korea	2015
55	KT806011	Saudi Arabia	2015
56	MG011360	Saudi Arabia	2017
57	KF186566	Saudi Arabia	2013
58	KT805975	Saudi Arabia	2015
59	MH310909	Saudi Arabia	2017
60	KF600628	Saudi Arabia	2013
61	KM027262	Saudi Arabia	2014
62	KJ829365	USA	2014
63	KP236092	United Arab Emirates	2013
64	KT121579	Saudi Arabia	2014
65	KT805970	Saudi Arabia	2015
66	KT806016	Saudi Arabia	2015
67	KT806036	Saudi Arabia	2015
68	KT806049	Saudi Arabia	2015
69	KT868871	South Korea	2015
70	MG912607	Saudi Arabia	2017
71	MG011356	Saudi Arabia	2016
72	KT806054	Saudi Arabia	2015
73	KT805968	Saudi Arabia	2015
74	KF961221	Qatar	2013
75	KT182956	South Korea	2015
76	KJ156952	Saudi Arabia	2013
77	KT806055	Saudi Arabia	2015
78	KX034095	South Korea	2015
79	KJ361503	France	2013
80	KJ156944	Saudi Arabia	2013
81	MF741830	Jordan	2015
82	KT805981	Saudi Arabia	2015
83	KT806008	Saudi Arabia	2015
84	MG912597	Saudi Arabia	2017
85	KM027257	Saudi Arabia	2014
86	KX154693	Saudi Arabia	2016
87	KJ156876	Saudi Arabia	2013
88	KT806037	Saudi Arabia	2015
89	KT805966	Saudi Arabia	2015
90	KJ361499	France	2013
91	MG912609	Saudi Arabia	2017
92	KT374057	South Korea	2015
93	KY581694	United Arab Emirates	2014
94	KM027268	Saudi Arabia	2014
95	KM027274	Saudi Arabia	2014
96	MH310912	Saudi Arabia	2017

Serial	Genbank Accession No.	Country	Year of Isolation
97	KT806020	Saudi Arabia	2015
98	MG757605	Saudi Arabia	2016
99	KP209310	United Arab Emirates	2014
100	MG757597	Saudi Arabia	2015
101	KF600613	Saudi Arabia	2013
102	KT805994	Saudi Arabia	2015
103	KF600620	Saudi Arabia	2012
104	MG011354	Saudi Arabia	2016
105	KT805988	Saudi Arabia	2015
106	KT225476	Thailand	2015
107	KT806003	Saudi Arabia	2015
108	KF600626	Saudi Arabia	2013
109	KF600634	Saudi Arabia	2013
110	KT806038	Saudi Arabia	2015
111	KT806041	Saudi Arabia	2015
112	KF811036	Tunisia	2013
113	KM027277	Saudi Arabia	2014
114	KM027285	Saudi Arabia	2014
115	KT806028	Saudi Arabia	2015
116	MG520075	Saudi Arabia	2015
117	MH454272	Saudi Arabia	2016
118	KR912188	Saudi Arabia	2014
119	KT357810	Saudi Arabia	2014
120	MG546330	Saudi Arabia	2015
121	KT805983	Saudi Arabia	2015
122	KT805991	Saudi Arabia	2015
123	KM210277	United Kingdom	2013
124	KX154685	Saudi Arabia	2016
125	KT868872	South Korea	2015
126	KY673148	Oman	2015
127	KR011266	Saudi Arabia	2014
128	KM027276	Saudi Arabia	2014
129	KF600652	Saudi Arabia	2012
130	KM027286	Saudi Arabia	2014
131	KC776174	Jordan	2012
132	KJ361502	France	2013
133	KY581684	United Arab Emirates	2013
134	KT374051	South Korea	2015
135	KT806015	Saudi Arabia	2015
136	KT861632	Jordan	2014
137	KT374056	South Korea	2015
138	KT806022	Saudi Arabia	2015
139	KJ156905	Saudi Arabia	2013
140	KT806032	Saudi Arabia	2015
141	KR912196	Saudi Arabia	2014
142	KX034097	South Korea	2015
143	KM027284	Saudi Arabia	2014
144	MG366881	Saudi Arabia	2015
145	KT156561	Oman	2013
146	KX154694	Saudi Arabia	2016
147	KJ156936	Saudi Arabia	2013
148	KM027273	Saudi Arabia	2014

Serial	Genbank Accession No.	Country	Year of Isolation
149	KJ156911	Saudi Arabia	2013
150	KJ156869	Saudi Arabia	2013
151	MG011362	Saudi Arabia	2016
152	KC164505	United Kingdom	2012
153	KX154690	Saudi Arabia	2016
154	KX034093	South Korea	2015
155	KT805976	Saudi Arabia	2015
156	KF600630	Saudi Arabia	2015
157	KJ361501	France	2013
158	KX154692	Saudi Arabia	2016
159	KR912191	Saudi Arabia	2014
160	MH395139	Saudi Arabia	2016
